# Heating quinoa shoots results in yield loss by inhibiting fruit production and delaying maturity

**DOI:** 10.1111/tpj.14699

**Published:** 2020-02-24

**Authors:** Jose C. Tovar, Carlos Quillatupa, Steven T. Callen, S. Elizabeth Castillo, Paige Pearson, Anastasia Shamin, Haley Schuhl, Noah Fahlgren, Malia A. Gehan

**Affiliations:** ^1^ Donald Danforth Plant Science Center St. Louis MO 63132 USA; ^2^ Bayer US – Crop Science St. Louis MO 63141 USA

**Keywords:** heat, yield, quinoa, fruit production, plant maturity, phenomics, RNA‐seq

## Abstract

Increasing global temperatures and a growing world population create the need to develop crop varieties that provide higher yields in warmer climates. There is growing interest in expanding quinoa cultivation, because of the ability of quinoa to produce nutritious grain in poor soils, with little water and at high salinity. The main limitation to expanding quinoa cultivation, however, is the susceptibility of quinoa to temperatures above approximately 32°C. This study investigates the phenotypes, genes and mechanisms that may affect quinoa seed yield at high temperatures. Using a differential heating system where only roots or only shoots were heated, quinoa yield losses were attributed to shoot heating. Plants with heated shoots lost 60–85% yield as compared with control plants. Yield losses were the result of lower fruit production, which lowered the number of seeds produced per plant. Furthermore, plants with heated shoots had delayed maturity and greater non‐reproductive shoot biomass, whereas plants with both heated roots and heated shoots produced higher yields from the panicles that had escaped the heat, compared with the control. This suggests that quinoa uses a type of avoidance strategy to survive heat. Gene expression analysis identified transcription factors differentially expressed in plants with heated shoots and low yield that had been previously associated with flower development and flower opening. Interestingly, in plants with heated shoots, flowers stayed closed during the day while the control flowers were open. Although a closed flower may protect the floral structures, this could also cause yield losses by limiting pollen dispersal, which is necessary to produce fruit in the mostly female flowers of quinoa.

## Significance Statement

This study provides evidence that heating quinoa during flowering results in seed yield loss by lowering fruit production. Plants with low yield after heat treatment also matured more slowly, suggesting that quinoa may use a type of avoidance strategy to survive heat stress conditions. Genes differentially expressed under heat include genes involved in flower development and flower opening.

## Introduction

Global temperatures are estimated to increase by 1–5°C (Callery *et al.*, 2018), while the world population will grow by approximately 47% in the 21st century (United Nations, 2017). On average, more than half of human caloric intake comes directly from grain consumption (Awika, 2011), and the feed used for meat production is 28% grain (Herrero *et al.*, 2013). Thus, there is a general need to increase grain production in a warming environment. Although farming technology, farmland expansion and breeding have increased absolute grain yields, the percentage yield gains for grain crops have been decreasing in recent years (Grassini *et al.*, 2013). Grain crops are predicted to lose 3.1–7.4% yield for every 1°C increase unless new varieties are developed for warmer temperatures (Zhao *et al.*, 2017). Climate change‐induced increases in temperature and reductions in precipitation have already caused yield losses of up to 5.5% from grain crops between 1980 and 2008 (Lobell *et al.*, 2011), a period in which temperatures increased by only approximately 0.6°C (Hansen *et al.*, 2010). To meet future global grain demands, it is vital to develop grain crop varieties that are adapted to higher temperatures (Challinor *et al.*, 2014) and to gain a better understanding of the molecular mechanisms that influence losses in grain yield.

Quinoa (*Chenopodium quinoa* Willd.) is a grain crop (pseudocereal) grown in areas with average temperatures of 9–30°C (Bhargava *et al.*, 2007a). There is growing interest in expanding quinoa cultivation (Jacobsen, 2003; Pulvento *et al.*, 2010; Choukr‐Allah *et al.*, 2016; Bazile *et al.*, 2016; Maliro *et al.*, 2017), because of its nutritious grain (Repo‐Carrasco *et al.*, 2003; Vega‐Gálvez *et al.*, 2010; Choukr‐Allah *et al.*, 2016) and its ability to grow on poor soils (Jacobsen *et al.*, 2003). Heat is a major limitation to expanding quinoa cultivation (Lesjak and Calderini, 2017; Hinojosa, Matanguihan, *et al.*, 2018), however, and quinoa generally does poorly in climates with average temperatures higher than 32°C (Bazile *et al.*, 2016; Hinojosa, Matanguihan, *et al.*, 2018). A goal of this study is to gain a better understanding of how heat limits grain production in quinoa.

The ability of quinoa to grow on poor soils and produce nutritious grain suggests that quinoa could be an interesting crop for the study of nutrient uptake. We are interested in how heat stress affects the processes of nutrient uptake in the roots and grain production in the shoots, as there are few studies on how roots and shoots differentially respond to heat stress. Previous studies have shown that plants respond differently to heat in the roots compared with heat in the shoots (Heckathorn *et al.*, 2013). In wheat, root heating had a more pronounced effect than shoot heating on grain yield, shoot biomass and root biomass (Kuroyanagi and Paulsen, 1988). In the grass *Agrostis palustris, *photosynthesis was more severely affected when roots were heated than when shoots were heated (Xu and Huang, 2000; Huang *et al.*, 2001). These studies suggest that the root and the shoot responses to heat may involve different mechanisms. Thus, studying how quinoa roots and shoots respond to heat can provide important insights into the mechanisms involved in yield losses.

This study investigates the phenotypic and transcriptomic responses of quinoa to root heating compared with shoot heating in the sequenced accession QQ74 (PI 614886; Jarvis *et al.*, 2017). The impact of heat on plant development, yield and gene expression are examined in detail, and we propose a possible mechanism for yield loss under heat stress.

## Results and Discussion

### Shoot heating significantly decreases yield, but root heating does not have a substantial effect on yield

To study the responses of quinoa to root and shoot heat stress, a sandbox system was designed and built that allowed for the independent temperature control of the roots and shoots (Figure S1). As previous studies have indicated that flowering is the most susceptible developmental stage to heat stress (Lesjak and Calderini, 2017), we heat‐treated quinoa during the flowering stage (in plants approx. 35–40 days old). Previous studies in both growth chambers and in the field showed that temperatures above 32°C impaired the yield and caused physiological and phenotypic changes in quinoa (Bazile *et al.*, 2016; Hinojosa, Matanguihan, *et al.*, 2018), and therefore the growth chamber temperature was set to 35°C. We found that the soil temperature was 30°C when the air temperature was 35°C, and therefore 30°C was selected as the heat treatment for roots. In total there were four treatments: (i) control treatment, with roots and shoots held at 22°C; (ii) heated roots (HR), with roots held at 30°C and shoots held at 22°C; (iii) heated shoots (HS), with shoots held at 35°C and roots held at 22°C; and (iv) heated roots and shoots (HRS), with roots at held at 30°C and shoots held at 35°C. Flowering plants were heat treated for 11 days and then returned to control temperature conditions until harvest. For further details of the sandbox set‐up, please see 'Plant material and growth conditions' in the Experimental procedures section.

Data analysis was performed using a factorial design, to quantify the effects of root versus shoot heating. A treatment‐by‐treatment comparison was performed to identify pairwise differences. For more details on the methods used for data analysis, please see 'Statistical analysis' in the Experimental procedures section.

**Figure 1:**
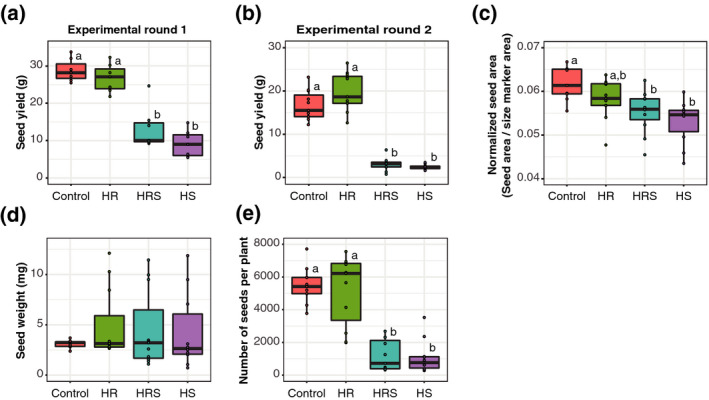
Yield analysis. (a) Yield per plant for each treatment in experimental round 1 (*n* = 15 plants). (b) Yield per plant for each treatment in experimental round 2 (*n* = 15 plants). (c) Average normalized seed area per plant for each treatment (*n* = 15 plants). (d) Average estimated individual seed weight per plant for each treatment (*n* = 15 plants). (e) Estimated number of seeds produced per plant for each treatment (*n* = 15 plants). Letters above boxes represent statistical significance at *P* < 0.05 from a Wilcoxon rank sum test.

In this study, yield was defined as grams of seed produced per plant. Shoot heating resulted in significant seed yield loss (two‐way analysis of variance, anova, on the effect of shoot heating, *P* < 0.0001). HS plants produced an average of 68% less seed yield relative to control plants (Wilcoxon rank sum test, *P* = 0.0043), and HRS plants had 61% less seed yield than control plants (Wilcoxon rank sum test, *P* = 0.0043; Figure 1a). Interestingly, root heating did not have a substantial effect on seed yield (two‐way anova on the effect of root heating, *P* = 0.9658), with HR plants showing only a 4% median difference from control plants (Wilcoxon rank sum test, *P* = 0.938). An experimental replicate of total plant yield showed similar yield losses, with significantly lower seed yield compared with the control (two‐way anova on the effect of shoot heating, *P* < 0.0001) and with no substantial effect from root heating (two‐way anova on the effect of root heating, *P* = 0.0856). In the second experimental replicate, plants with HS had an average of 85% less yield than control plants (Wilcoxon rank sum test, *P* = 0.000021), and plants with HRS produced an average of 81% less yield than control plants (Wilcoxon rank sum test, *P* = 0.000021). In contrast, plants with HR had a 20% median yield difference from control plants (Wilcoxon rank sum test, *P* = 0.2957; Figure 1b). Overall, these results indicate that heating quinoa shoots results in much greater yield losses than heating roots.

Although no other quinoa studies have looked at the differential effects of heat on quinoa roots and shoots, previous studies on quinoa have found varied yield responses to heat. Quinoa accession Regalona lost 31% yield, whereas accession BO5 lost 23% yield when night temperatures were 22°C instead of 18°C (control) during flowering (Lesjak and Calderini, 2017; Hinojosa, González, *et al.*, 2018). Hinojosa *et al.*, 2018 found that quinoa accessions QQ74 (used in this study) and 17GR did not lose any yield when treated at 40°C day/24°C night during flowering, as compared with the control (22°C day/16°C night) (Hinojosa, Matanguihan, *et al.*, 2018). The differences in QQ74 yield under heat in this study compared with Hinojosa, Matanguihan, *et al. *(2018) may result from the methodology used for the treatment, including the temperatures used and the duration of the treatment. One similarity between studies that found yield losses under heat stress (this study; Lesjak and Calderini, 2017; Hinojosa, González, *et al.*, 2018) was a high night‐time temperature treatment during flowering. Similarly, in *Triticum aestivum* (wheat) and *Oryza sativa* (rice), high‐temperature treatments at night have been shown to negatively impact yield (Shi *et al.*, 2013; Narayanan *et al.*, 2015; Jagadish *et al.*, 2015; Shi *et al.*, 2016).

### Yield losses from shoot heating are mainly the result of a smaller number of seeds produced

To investigate the changes in overall seed yield, we examined whether yield losses were largely the result of changes in seed size or seed number. We analyzed seed size by two independent measurements: (i) average seed weight and (ii) average seed area (measured from images). Seed number was estimated by dividing the total seed weight by the average seed weight for each plant. For more information on how seed size and seed number were measured, please see 'Main panicle, seed and whole plant imaging' in the Experimental procedures section.

Shoot heating had a significant effect on seed area (two‐way anova on the effect of shoot heating, *P* = 0.0455), whereas root heating did not show a substantial effect, with only a 5% median difference from the control (two‐way anova on the effect of root heating, *P* = 0.708). HS plants had 14% smaller seed area than control plants (Wilcoxon rank sum test, *P* = 0.0095; Figure 1c) and HRS plants had 11% smaller seed area than control plants (Wilcoxon rank sum test, *P* = 0.0312; Figure 1c). Similarly, Bertero *et al.*, 1999 reported a 14% smaller seed diameter with heat treatment in the quinoa accession Kancolla (Bertero *et al.*, 1999). Although the seed area was significantly smaller in our shoot‐heated samples when compared with controls, the estimated individual seed weight was not substantially affected by heat, with median values differing only 1–19% from the control values (two‐way anova on the effects of root and shoot heating, *P* > 0.05; Figure 1d). The decrease in seed area without substantial changes to individual seed weight with heat treatment is similar to that reported in previous work. For example, in quinoa accession Regalona, night‐time heat treatment (22°C) resulted in yield losses without substantial changes in average seed weight (Lesjak and Calderini, 2017). Similar to our study, Hinojosa *et al.*, 2018 also found that seed weight was not substantially affected by heat in accession QQ74 (Hinojosa, Matanguihan, *et al.*, 2018).

The number of seeds produced per plant was estimated by dividing the total yield (g) by the individual seed weight (for details, please see 'Main panicle, seed, and whole plant imaging' in the Experimental procedures). There was no substantial difference in the estimated individual seed weight with heat treatment, but there was a significant decrease in the total seed yield, which suggests a change in seed number. Accordingly, there was a significant effect of shoot heating on seed number (two‐way anova on the effect of shoot heating, *P* < 0.0001), but no substantial effect from root heating on seed number (6% average difference from control, two‐way anova on the effect of root heating, *P* = 0.5933). The estimated seed number was an average of approximately 79% lower in the HS treatment than in the control (Wilcoxon rank sum test, *P* = 0.00016) and an average of approximately 78% lower in the HRS treatment than in the control (Wilcoxon rank sum test, *P* = 0.00016; Figure 1e). Overall, there were fewer seeds produced per plant and less seed area but no substantial difference in individual seed weight between plants with heated shoots and control plants. This indicates that the observed yield losses in plants with heated shoots are mainly the result of fewer seeds produced.

### Main and secondary panicles from plants with heated shoots had less yield than those from control plants; tertiary panicles from HRS plants had higher yield than control tertiary panicles

**Figure 2:**
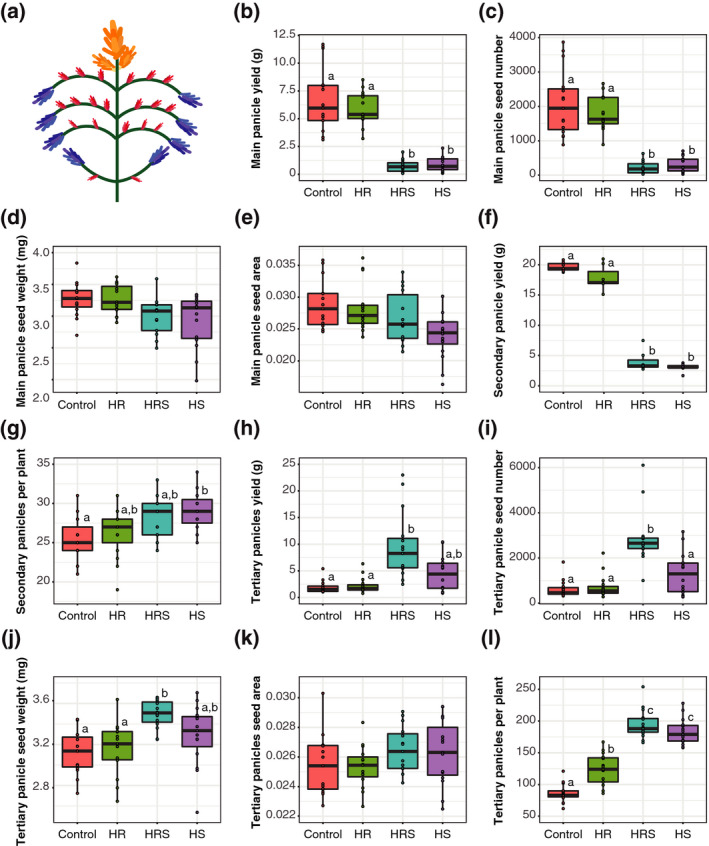
Yield analysis by panicle type. (a) Locations of the main panicle (the first panicle to emerge; orange), secondary panicles at the tip of each branch (emerging after the main panicle; blue) and tertiary panicles from nodes within branches (emerging after the secondary panicles; red). (b) Yield from the main panicle of each plant for each treatment (*n* = 15). (c) Number of seeds produced per main panicle for each treatment (*n* = 15). (d) Average seed weight per main panicle for each treatment (*n* = 15). (e) Average main panicle normalized seed area per plant for each treatment (*n* = 15). (f) Aggregated yield from all secondary panicles in each plant, for each treatment (*n* = 15). (g) Number of secondary panicles produced by each plant for each treatment (*n* = 15). (h) Total tertiary panicle yield per plant for each treatment (*n* = 15). (i) Number of seeds produced from all tertiary panicles in each plant for each treatment (*n* = 15). (j) Average tertiary panicle seed weight per plant for each treatment (*n* = 15). (k) Average normalized tertiary panicle seed area from each plant and for each treatment (*n* = 15). (l) Number of tertiary panicles produced by each plant for each treatment (*n* = 15). Control, roots and shoots held at 22°C; HR, heated roots, with roots held at 30°C and shoots held at 22°C; HS, heated shoots, with shoots held at 35°C and roots held at 22°C; HRS, heated roots and shoots, with roots at held at 30°C and shoots held at 35°C. Letters above boxes represent statistical significance at *P* < 0.05 from a Wilcoxon rank sum test.

Quinoa yield is the total seed production from many panicles on each plant. Therefore, we further examined where the yield losses were occurring on each plant (i.e. in primary, secondary or tertiary panicles; Figure 2a). The 11‐day heat treatment was started at first anthesis of the main panicle, but the panicles produced by a quinoa plant emerge and develop at different times. Therefore, the heat treatment was applied to panicles at different developmental stages and for different durations. To assess the contributions of different panicle types to yield, quinoa panicles were classified into three groups (Figure 2a): (i) the main panicle – the first panicle to emerge, at the top of the plant; (ii) secondary panicles – the panicles at the tip of each branch, emerging after the main panicle; and (ii) tertiary panicles – the panicles originating from nodes within branches, emerging after the secondary panicle in the same branch.

The main panicle yield was significantly affected by shoot heating (two‐way anova on the effect of shoot heating, *P* < 0.0001), but root heating did not show substantial effects (10% median difference from control, two‐way anova on the effect of root heating, *P* = 0.6953). Main panicles from HS plants yielded 87% less than control plants (Wilcoxon rank sum test, *P* = 0.00017; Figure 2b). Similarly, main panicles from HRS plants yielded 89% less than control plants (Wilcoxon rank sum test, *P* = 0.00019; Figure 2b). Based on image analysis, the losses in the main panicle yield from shoot heating were caused by a reduction in the number of seeds produced (Figure 2c). The main panicle from HRS plants produced 89% fewer seeds than the control plants (Wilcoxon rank sum test, *P* < 0.0001) and the main panicle from HS plants produced 85% fewer seeds than control plants (Wilcoxon rank sum test, *P* < 0.0001). The seed number from main panicles of HR plants did not substantially differ from that in control plants (13% average difference from control, Wilcoxon rank sum test, *P* = 0.97). The main panicle seed weight (Figure 2d) and area (Figure 2e) were not substantially changed by heat treatments (2–6% median differences from control, Wilcoxon rank sum test, *P* > 0.05).

Like the main panicle yield, the secondary panicle yield was also affected by shoot heating (two‐way anova on the effect of shoot heating, *P* < 0.0001), with root heating not showing substantial effects (9% average difference from control, two‐way anova on the effect of root heating, *P* = 0.1801). The average yield of all the secondary panicles from each HS plant was 85% lower than in control plants (Wilcoxon rank sum test, *P* = 0.0021; Figure 2f), despite producing four more secondary panicles on average than the control plants (Wilcoxon rank sum test, *P* = 0.021; Figure 2g). HRS plants produced an average of 79% less yield from secondary panicles than in control plants (Wilcoxon rank sum test, *P* = 0.0021; Figure 2f). Although HRS plants produced a median of four more secondary panicles per plant than control plants, the statistical analysis for HRS did not show a clear effect (Wilcoxon rank sum test, *P* = 0.067). Thus, the results suggest that yield losses from secondary panicles were largely caused by shoot heating, similar to main panicles. The relationship between shoot heating and secondary panicle number is less clear, however.

Unlike main and secondary panicles, tertiary panicles emerged after the heat treatment ended. All heat‐treated plants had more tertiary panicles than control plants. HR plants produced approximately 43% more tertiary panicles than control plants (Wilcoxon rank sum test, *P* = 0.00026; Figure 2l). HRS plants (Wilcoxon rank sum test, *P* = 0.00002) and HS plants (Wilcoxon rank sum test, *P* = 0.00002) produced more than double the number of tertiary panicles than control plants (Figure 2l). Despite the increase in tertiary panicle number for all heat treatments, the increased tertiary panicle yield was only statistically significant in HRS plants, and not in HR or HS plants. The tertiary panicles of HRS plants had fivefold more yield than control tertiary panicles (Wilcoxon rank sum test, *P* = 0.0000057; Figure 2h). The average tertiary panicle yield from HS plants was fourfold higher than that of the control but was not statistically significantly different from that of the control (Wilcoxon rank sum test, *P* = 0.18). The average tertiary panicle yield from HR plants was not substantially different from the control (19% average difference from control, Wilcoxon rank sum test, *P* = 0.7; Figure 2h). Therefore, all heat‐treated plants produce more tertiary panicles, but the HRS treatment was the only treatment that produced significantly more tertiary panicle yield in comparison with control plants.

Based on image analysis, the higher yield of tertiary panicles observed in HRS plants resulted from a median 12% higher individual seed weight than in control plants (Wilcoxon rank sum test, *P* < 0.0001; Figure 2j), as well as a median 5.7‐fold higher number of seeds produced (Wilcoxon rank sum test, *P* < 0.0001; Figure 2i), but not to changes in seed area (only 4% median difference from control, Wilcoxon rank sum test, *P* > 0.05; Figure 2k). The increased tertiary panicle yield and seed weight in HRS plants suggests that more resources are allocated to tertiary panicles compared with controls. This reallocation of resources to tertiary panicles in HRS plants cannot compensate for yield losses in the main and secondary panicles, however, because HRS plants still produced a lower total yield than the control plants.

### Secondary panicle yield was significantly affected by shoot heating and panicle position in the plant, but the length of heat treatment did not show a substantial effect

**Figure 3:**
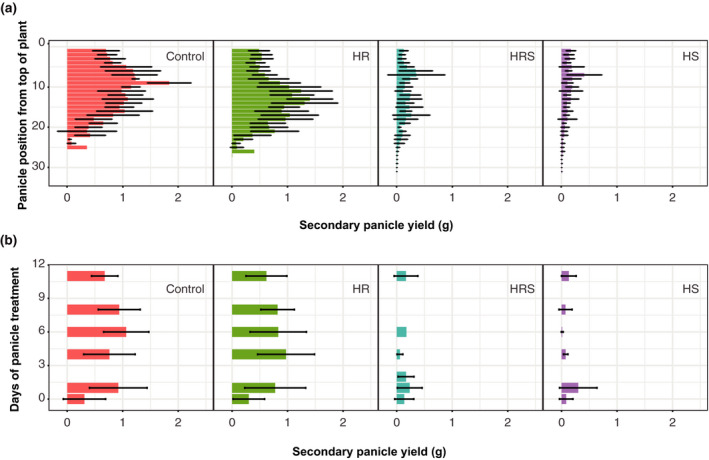
Analysis of the yield of secondary panicles. (a) Yield of individual secondary panicles by position in the plant for each treatment (*n* = 1–7 secondary panicles, depending on position). (b) Yield from individual secondary panicles heat treated for 0, 1, 2, 4, 6, 8 or 11 days: *n* = 9 (control), 22 (HR), 86 (HRS) and 72 (HS) panicles for 0 days; *n* = 46 (control), 21 (HR), 6 (HRS) and 4 (HS) panicles for 1 day; *n* = 8 (HRS) panicles for 2 days; *n* = 19 (control), 34 (HR), 5 (HRS) and 3 (HS) panicles for 4 days; *n* = 59 (control), 36 (HR), 1 (HRS) and 6 (HS) panicles for 6 days; *n* = 9 (control), 11 (HR) and 6 (HS) panicles for 8 days; *n* = 10 (control), 48 (HR), 79 (HRS) and 101 (HS) panicles for 11 days. Control, roots and shoots held at 22°C; HR, heated roots, with roots held at 30°C and shoots held at 22°C; HS, heated shoots, with shoots held at 35°C and roots held at 22°C; HRS, heated roots and shoots, with roots at held at 30°C and shoots held at 35°C. Error bars represent standard deviation.

Shoot heating significantly affected the overall yield from all secondary panicles in a quinoa plant; however, secondary panicles from a single quinoa plant emerged and matured at different times, creating differences in their exposure to heat treatment. To assess whether yield was affected by different exposure lengths to heat, the yield and time of heat exposure of each secondary panicle was recorded. The distribution of yield among secondary panicles was analyzed through factorial analysis to study the effects of: (i) shoot heating; (ii) root heating; (iii) secondary panicle position in the plant; and (iv) days of heat treatment since secondary panicles showed visible anthers. Panicles in quinoa start emerging from the top of the plant. Therefore, secondary panicles near the top of the plant mature earlier than panicles near the bottom. Factors three and four were analyzed in separate anova s because they were not independent variables. Main panicles were heat treated from first anthesis; however, anthesis could not be used to measure days of heat exposure in secondary panicles, because anthesis of the secondary panicles was inhibited in plants with heated shoots. Therefore, the days of heat treatment since anthers were visible in the secondary panicle were used to measure the effects of heat duration on yield. Secondary panicle yield was affected by shoot heating (three‐way anova on the effect of shoot heating, *P* < 0.0001) and position of the secondary panicle (three‐way anova on the effect of panicle position, *P* < 0.0001; Figure 3a). Interestingly, the length of heat treatment did not have a substantial effect on secondary panicle yield, with yield losses being similar in magnitude regardless of how long the panicles were treated (three‐way anova effect of days of heat treatment since visible anthers, *P* = 0.13034; Figure 3b). This indicates that a short heat exposure during anthesis may be sufficient to cause significant yield losses.

### Shoot heating results in significantly fewer flowers developing fruit

**Figure 4:**
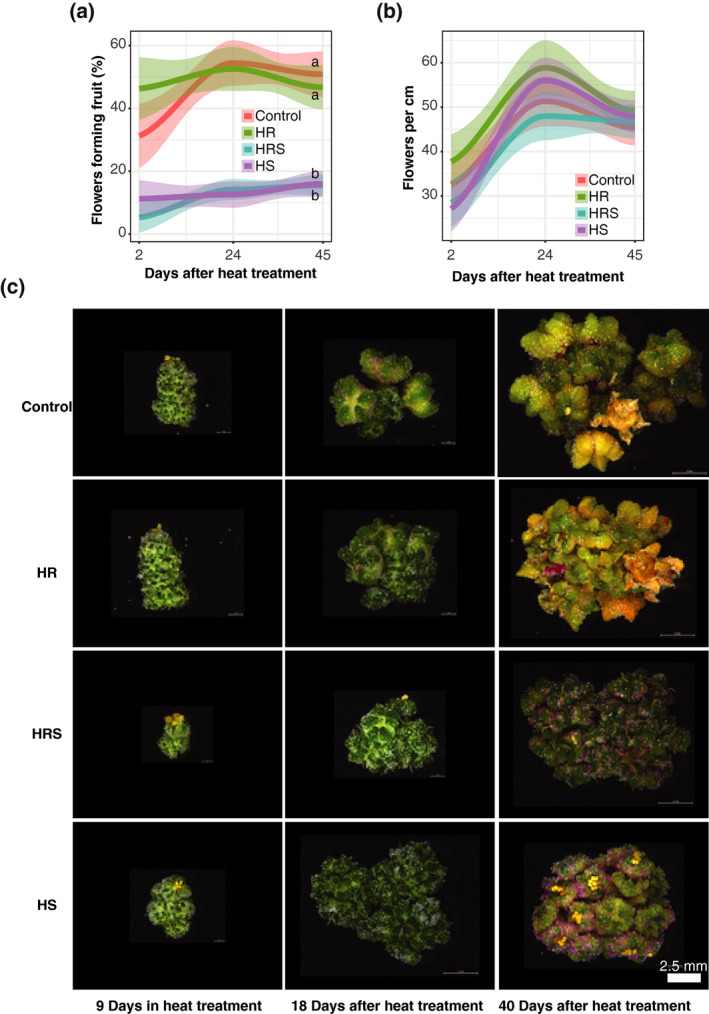
Main panicle flower analysis. (a) Fruit production measured on days 2, 24 and 45 after heat treatment ended. (b) Flower density in the most apical 5 cm of the main panicle measured on days 2, 24 and 45 after heat treatment ended. Curves resulting from a LOESS (locally estimated scatter‐plot smoothing) polynomial regression are shown (*n* = 30 main panicles, for each time point and each treatment). Letters next to curves represent statistical significance at *P* < 0.05 from a Kolmogorov–Smirnov test. (c) Flowers from the main panicle for each treatment imaged after 9 days of heat treatment, 18 days after heat treatment ended and 40 days after heat treatment ended. All images have been scaled to the same scale bar.

To investigate the cause of reduced seed number in HS and HRS plants, flower development in the main panicle was observed during and after heat treatment. After 11 days of heat treatment, all plants were grown at control temperature until harvest. At 2, 24 and 45 days after the heat treatment ended, the total number of flowers and flowers bearing fruit in the apical 5 cm of the main panicle were counted. Fruit production was significantly lower in HS and HRS plants than in control plants (Kolmogorov–Smirnov test, *P* < 0.00001), whereas root heating did not have a substantial effect on fruit production (12% average difference from control, Kolmogorov–Smirnov test, *P* = 0.09956, Figure 4a). There was a significant effect of shoot heating on fruit production at all time points measured (2, 24 and 45 days after heat treatment ended; two‐way anova *P* < 0.00001). HS plants produced approximately 35.9% of the fruit produced by control plants at 2 days after heat treatment ended (Wilcoxon rank sum test, *P* = 0.02093), approximately 23.1% at 24 days after heat treatment ended (Wilcoxon rank sum test, *P* < 0.00001), and approximately 31.4% at 45 days after heat treatment ended (Wilcoxon rank sum test, *P* < 0.00001). HRS plants produced approximately 16.8% of the fruit produced by control plants at 2 days after heat treatment ended (Wilcoxon rank sum test, *P* = 0.00079), approximately 26% at 24 days after heat treatment ended (Wilcoxon rank sum test, *P* < 0.00001), and approximately 30.5% at 45 days after heat ended (Wilcoxon rank sum test, *P* < 0.00001). Overall, fruit production was significantly reduced in treatments with yield losses.

Along with fewer flowers producing fruit, another possible contributor to seed yield loss is a lower density of flowers along the panicle. To investigate the possibility that shoot heating lowers flower density in a quinoa plant, all the flowers in the apical 5 cm of the main panicle were counted. The number of flowers being produced in the apical 5 cm across treatments was similar across treatments (Kolmogorov–Smirnov test, *P*> 0.05; Figure 4b). Although this measurement does not discount the possibility that there are differences in flower concentration in lower regions of the plant, it does suggest that flower concentration in the main panicle is similar between treatments.

To assess possible differences in fruit production and flower development, flowers from the most apical part of the main panicle were observed under the microscope with 9 days of heat treatment, 18 days after heat treatment ended and 40 days after heat treatment ended. All flowers appeared similar among all treatments with 9 days of heat treatment; however, 18 days after the heat treatment ended, several flowers with fruit were visible in both control and HR treatments, but little to no fruit was observed in HS and HRS samples (Figure 4c). At 40 days after heat treatment ended, seed was formed in both control and HR plants, but not in HS or HRS plants (Figure 4c). In general, flowers of HS and HRS plants appeared less developed than control and HR flowers and lacked fruit and seed production (Figure 4c). The lack of fruit production and differences in flower development may explain the observed low seed number and yield loss in treatments with heated shoots.

### Photosynthesis and pollen viability with heat treatment were similar to controls during and after heat treatment

As our study observed a significant decrease in yield and fruit production in response to heat stress, we aimed to better understand the physiological changes occurring during heat stress that may have contributed to yield losses. We hypothesized that photosynthesis and pollen viability under heat treatment are negatively impacted. Although changes in photosystem‐II efficiency have been associated with grain yield (Ort *et al.*, 2015; Sanchez‐Bragado *et al.*, 2016; Lin *et al.*, 2018), as well as with responses to heat (Mathur and Jajoo, 2014; Yamamoto, 2016), we did not find statistically significant evidence that photosystem‐II efficiency was affected by heat. Similarly, we did not find statistically significant changes in pollen viability, despite pollen viability having been reported to be affected by heat in other species (Schoper *et al.*, 1987; Vara Prasad *et al.*, 1999; Kumar *et al.*, 2016; Jiang *et al.*, 2019), as well as in quinoa (Hinojosa, Matanguihan, *et al.*, 2018). Thus, we cannot conclude that either photosystem‐II efficiency or pollen viability were significant factors in yield losses after heat treatment (please see supplemental materials for data and details; Figures S2 and S3).

### Main panicles had less area and had a more open structure with heated shoot treatment

**Figure 5:**
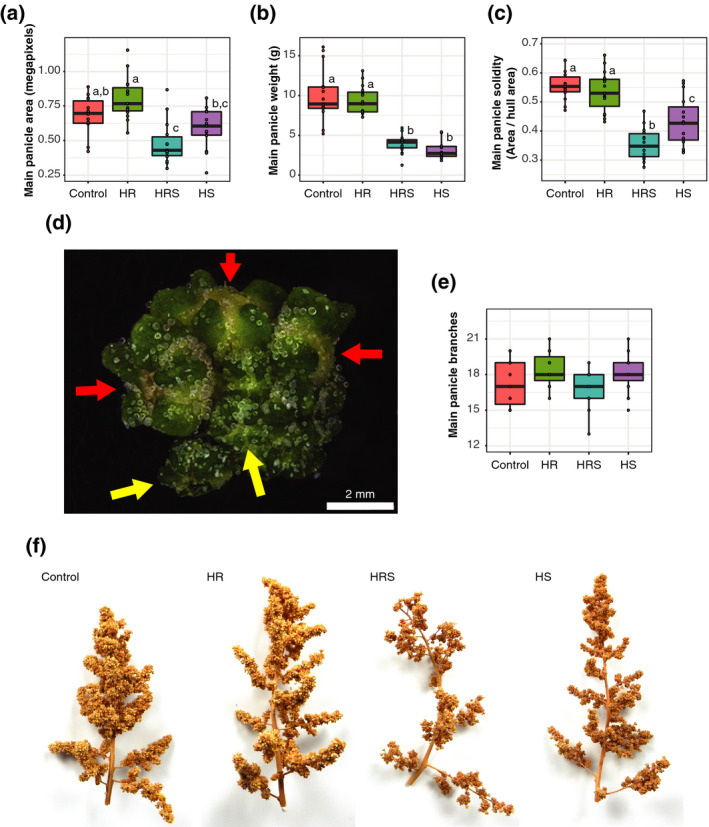
Analysis of main panicle structure. (a) Area of each main panicle at harvest per treatment (*n* = 15). (b) Weight of each main panicle at harvest for each treatment (*n* = 15). (c) Main panicle solidity for each plant and for each treatment (*n* = 15). (d) Segment of a quinoa main panicle showing fruit (red arrows) and unfertilized flowers (yellow arrows). (e) Number of branches in each main panicle for each treatment (*n* = 15). (f) Example images of the main panicle from each treatment group (representative median image for area) at harvest. Control, roots and shoots held at 22°C; HR, heated roots, with roots held at 30°C and shoots held at 22°C; HS, heated shoots, with shoots held at 35°C and roots held at 22°C; HRS, heated roots and shoots, with roots at held at 30°C and shoots held at 35°C. Letters above boxes represent statistical significance at *P* < 0.05 from a Wilcoxon rank sum test.

Next, we hypothesized that panicle architecture may be affected by heat. Although image analysis of the main panicles found no substantial difference in the height or width of the main panicle, the main panicle area (two‐way anova on the effect of shoot heating, *P* < 0.00001; Figure 5a) and the main panicle weight (two‐way anova on the effect of shoot heating, *P* < 0.00001; Figure 5b) were reduced after shoot heating relative to control plants. Reduced main panicle area and weight are likely to result from lower fruit production after shoot heating, as fruits are much larger than unfertilized flowers (Figure 5d), and the difference in fruit production from shoot heating was significant. Additionally, solidity, a measure of panicle compactness, was also significantly reduced after shoot heating relative to control plants (two‐way anova on the effect of shoot heating, *P* < 0.00001; Figure 5c). Reduced solidity could be caused by the main panicles of HS and HRS plants having a more open structure, or by the main panicles having fewer branches. To study the cause of reduced solidity, the number of branches in the main panicles was counted, and a similar number of branches was found between any treatment and control (0 to 1 branch median difference from control, Wilcoxon rank sum test, *P*> 0.05; Figure 5e). This indicates that the observed reduced solidity in plants with heated shoots is likely to result from the more open structure relative to control plants (Figure 5f). Altogether, we found a significant difference in the structure of panicles from heated shoot treatments, which is likely to be a reflection of differences in fruit production but not flower concentration under heat.

### Shoot heating increases the dry weight of non‐reproductive shoot biomass

**Figure 6:**
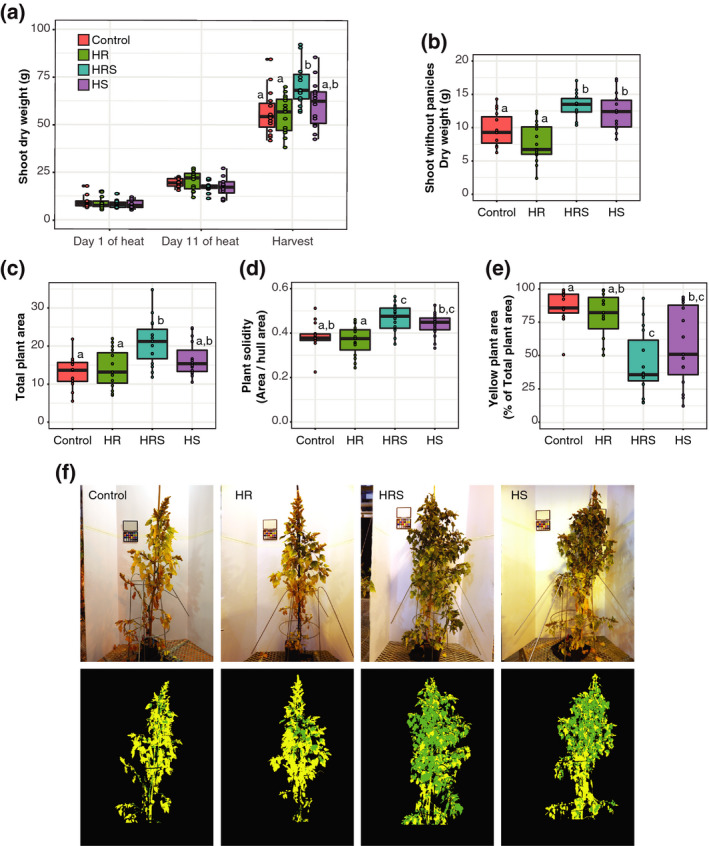
Shoot size and maturity. (a) Shoot dry weight (g) with 1 day of heat, 11 days of heat and at harvest (*n* = 9 for each treatment for days 1 and 11 of heat treatment, and *n* = 15 for each treatment at harvest). (b) Shoot dry weight without panicles at harvest (*n* = 15 for each treatment). (c) Normalized area for each plant and for each treatment (*n* = 15). (d) Solidity for each plant and for each treatment (*n* = 15). (e) Percentage yellow area for each treatment per plant (*n* = 15). (f) Example images from each treatment group (representative median image) classified into yellow or green pixels by naive Bayes classifier from plantcv. Control, roots and shoots held at 22°C; HR, heated roots, with roots held at 30°C and shoots held at 22°C; HS, heated shoots, with shoots held at 35°C and roots held at 22°C; HRS, heated roots and shoots, with roots at held at 30°C and shoots held at 35°C. Letters above boxes represent statistical significance at *P* < 0.05 from a Wilcoxon rank sum test.

Fruit development was significantly affected in HS and HRS treatments. Therefore, we hypothesized that there were differences in the development of non‐reproductive tissues with heat treatment. Quinoa shoot dry weight was measured on the first day of heat treatment, the last day of heat treatment and at harvest. Plant shoot fresh weight and root dry weight were similar across treatments on the first and last day of heat treatment (Figures S4 and S5). Plants from all treatments had similar shoot dry weight at the start of the heat treatment (5–14% average difference from control, Kruskal–Wallis test, *P* = 0.6227; Figure 6a), as well as after 11 days of heat treatment (5–12% average difference from control, Kruskal–Wallis test, *P* = 0.1787; Figure 6a). At harvest, HS plants (16% average difference from control, Wilcoxon rank sum test, *P* = 0.5929) and HR plants (2% average difference from control, Wilcoxon rank sum test, *P* = 1) had similar dry weights to control plants (Figure 6a); however, HRS plants had 27% greater shoot dry weight than control plants at harvest (Wilcoxon rank sum test, *P* = 0.0042). Interestingly, HRS plants also had significantly more tertiary panicles (Figure 2l) and tertiary panicle yield (Figure 2h), despite having significantly less total yield than control plants (Figure 1a,b).

Measurements of total shoot dry weight at harvest included the weight of panicles and seeds in addition to leaf and stem tissue. Therefore, to understand whether the quantity of non‐reproductive biomass was affected by heat, the shoot dry weight without panicles was also measured at harvest. The shoot dry weight without panicles was significantly affected by shoot heating (two‐way anova on the effect of shoot heating, *P* < 0.0001) but root heating did not show a substantial effect (11% average difference from control, two‐way anova on the effect of root heating, *P* = 0.4997). HS plants had 42% more dry weight in their shoots without panicles than control plants (Wilcoxon rank sum test, *P* = 0.03717), and HRS plants had 48% more dry weight in their shoots without panicles than control plants (Wilcoxon rank sum test, *P* = 0.00242, Figure 6b). Higher shoot dry weight at harvest after heat treatment has also been reported for the cultivars Red Head, Cherry Vanilla, Salcedo (Bunce, 2017) and Titicaca (Yang *et al.*, 2016). Interestingly, HS and HRS plants developed more non‐reproductive biomass than control plants after the heat treatment concluded. This suggests that heat triggered an irreversible change in quinoa development, and potentially in resource allocation. Furthermore, this change might have been induced by the lack of seed production, as seed formation has been shown to trigger plant senescence in other species (Noodén and Penney, 2001; Schippers *et al.*, 2015).

### Shoot heating delays plant maturity

There was more non‐reproductive biomass in HS and HRS plants; therefore, we hypothesized that plant maturation was delayed by shoot heat treatment. Analysis of plant images 45 days after heat treatment revealed that plant architecture was affected by shoot heating. In agreement with the manually measured shoot dry weight results, the plant area extracted from image data was 59.8% higher in HRS plants than in control plants (Wilcoxon rank sum test, *P* = 0.007; Figure 6c). Plants from both HS (12% median change from control, Wilcoxon rank sum test, *P* = 0.299) and HR (3% median change from control, Wilcoxon rank sum test, *P* = 1) treatments had similar areas to control plants (Figure 6c), which mirrors the manual shoot dry weight measurements. Plant solidity (compactness) was higher in shoot‐heated plants than in control plants (two‐way anova on the effect of shoot heating, *P* = 0.0001), whereas the solidity in root‐heated plants was similar to control plants (5% average difference from control, two‐way anova on the effect of root heating, *P* = 0.5822). Quinoa plants shed their leaves at maturity. Therefore, solidity could be a proxy measurement for maturity because solidity is likely to decrease when leaves are shed. Accordingly, lower solidity could indicate fewer leaves and a more mature plant. HRS plants had 21% average higher solidity than control plants (Wilcoxon rank sum test, *P* = 0.0122; Figure 6d). HS plants had 19% average higher solidity than control plants, but the difference was not substantial (Wilcoxon rank sum test, *P* = 0.0768). Thus, although the combined HRS treatment had a significant effect on solidity, the effect of shoot heat treatment alone (HS) on solidity was less clear (Figure 6d).

We also used color to quantify plant maturity, with 'yellow' being more mature and 'green' being less mature. The plant area was classified into green and yellow pixels using the naive bayes classifier in plantcv (Gehan *et al.*, 2017). Shoot heating had a significant effect on the percentage of yellow plant area (two‐way anova on the effect of shoot heating, *P* = 0.0001; Figure 6e). Root heating did not have a substantial impact on the percentage of yellow plant area (8% average difference from control, two‐way anova on the effect of root heating, *P* = 0.3425). HS plants had 57% yellow area and HRS plants had 44% yellow area, whereas HR plants had 80% yellow area and control plants had 87% yellow area, on average (Wilcoxon rank sum test *P* values: control versus HS, *P* = 0.0235; control versus HRS, *P* = 0.001; control versus HR, *P* =0.9965; Figures 6e,f). The decreased proportion of yellow plant area in treatments with heated shoots suggests a delay in maturity in comparison with control plants. Changes in maturity in shoot‐heated samples, especially HRS, in combination with increases in tertiary panicle yield and number suggest that this accession of quinoa uses a heat‐avoidance strategy rather than an escape strategy. In an escape strategy a plant attempts to finish its life cycle (often with early flowering) in response to adverse environmental conditions, whereas in an 'avoidance' strategy the plant development slows down (Shavrukov *et al.*, 2017).

### Differentially expressed genes in treatments with yield losses

To identify candidate genes involved in yield loss caused by heat in quinoa, gene expression was examined. Differentially expressed genes compared with the control treatment were identified by RNA‐seq analysis. Leaf samples were collected from all treatments during day 1 and day 11 (last day) of heat treatment. To facilitate the use of the expression data generated in this study, an r shiny application, quinoa heat data explorer, was developed as a community tool (for details, see supplemental data; http://shiny.datasci.danforthcenter.org/quinoa-heat/).

**Figure 7:**
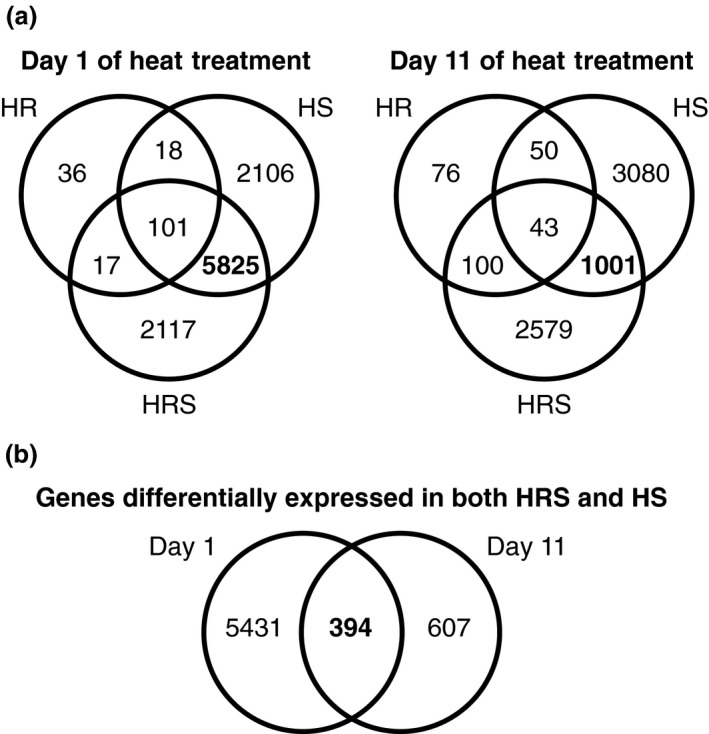
Differentially expressed genes by treatment and day of sampling. (a) Venn diagram of differentially expressed genes from control on day 1 of heat treatment and day 11 of heat treatment (*n* = 3 for each time point and each treatment). Overlapping genes in treatments with reduced yield are shown in bold. (b) Differentially expressed genes overlapping between day 1 and day 11 in treatments with reduced yield (HRS and HS).

To associate gene expression with yield loss, genes differentially expressed exclusively in the treatments with yield loss (HRS and HS) were analyzed further (Figure 7a). Day‐1 samples had 5825 differentially expressed genes in both HRS and HS treatments. Gene ontology (GO) over‐representation analysis of the 5825 differentially expressed genes found no over‐represented GO terms, as compared with GO term representation in the entire quinoa genome. Day‐11 samples had 1001 differentially expressed genes exclusively in both HRS and HS treatments, also with no GO terms over‐represented. Further analysis focused on the subset of 394 genes differentially expressed in both HRS and HS treatments and during both days 1 and 11 of heat treatment (Figure 7b). No GO terms were over‐represented among these 394 genes and panther GO enrichment analysis did not find any enriched GO terms in any of the gene lists.

### Transcription factor homologs show potential role of flower development on yield

A single transcription factor (TF) has the potential to modify the expression of many other genes, therefore differentially expressed transcription factors were identified in the 394 genes differentially expressed in HRS and HS treatments. Out of the 394 genes differentially expressed in both HRS and HS treatments during both day 1 and day 11 of heat treatment, 10 genes were identified as homologous to *Arabidopsis thaliana* transcription factors (Table S1). In the list of 10 transcription factors (Table S1), AUR62008425 is a homolog of *cdf3*, an Arabidopsis TF that affects flowering time under abiotic stress (Corrales *et al.*, 2017). Also from the list of 10 TFs (Table S1), AUR62019043, AUR62033383 and AUR62034763 are homologs of TFs associated with flower morphogenesis and development. AUR62019043 and AUR62033383 are homologs of *AGAMOUS‐LIKE 24* (*AGL24*) and *AGAMOUS‐LIKE 14* (*AGL14*), respectively, which affect flowering and flower development in Arabidopsis (Yu *et al.*, 2004; Liu *et al.*, 2007; Liu *et al.*, 2008; Torti and Fornara, 2012; Thouet *et al.*, 2012; Fernandez *et al.*, 2014; Pérez‐Ruiz *et al.*, 2015; Agliassa *et al.*, 2018). Furthermore, *AGL24* has been shown to be temperature responsive in Arabidopsis (Gregis *et al.*, 2006). AUR62034763 is associated with flower morphogenesis and development and is a homolog of the Arabidopsis TF *AUXIN RESPONSE FACTOR 2* (*ARF2*). *ARF2* is a pleiotropic developmental regulator (Okushima *et al.*, 2005) associated with flower opening, fertility and seed yield in Arabidopsis (Hughes *et al.*, 2008). The Arabidopsis *arf2* mutant makes sepals grow longer, preventing petals from separating, so the flowers do not open, which then lowers seed set and yield (Hughes *et al.*, 2008). As flower development was delayed in quinoa treatments with yield losses (HS and HRS; Figure 4c) and transcription factors differentially expressed in these treatments are associated with flower development, it is possible that flower development may have a significant role in yield losses with shoot heat treatments. Furthermore, it is possible that one or more of these genes associated with flowering time, flower development, flower fertility and flower opening may be involved in these yield losses.

### Shoot heating inhibits flower opening, potentially affecting pollination, fruit formation, and yield

**Figure 8:**
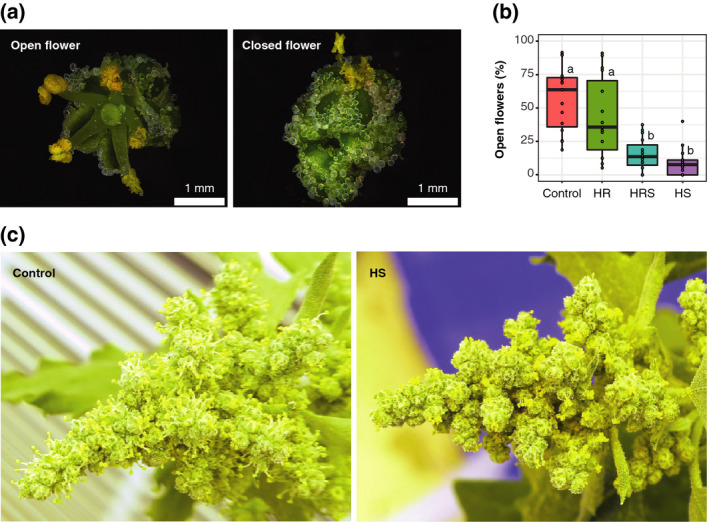
Shoot heating affected floral opening compared with the control. (a) Representative images of open and closed quinoa flowers. (b) Percentage of open hermaphrodite flowers in the main panicle on day 6 of heat treatment (*n* = 15). Control, roots and shoots held at 22°C; HR, heated roots, with roots held at 30°C and shoots held at 22°C; HS, heated shoots, with shoots held at 35°C and roots held at 22°C; HRS, heated roots and shoots, with roots at held at 30°C and shoots held at 35°C. Letters above boxes represent statistical significance at *P* < 0.05 from a Wilcoxon rank sum test. (c) Representative images of control and heated shoot main panicles.

The identification of genes associated with flower opening and flowering time in RNA‐seq experiments from leaf samples led us to examine flower opening and timing during heat treatment in quinoa. Quinoa inflorescences are composed of hermaphrodite and female flowers, where female flowers depend on the pollen from the hermaphrodite flowers to be fertilized, produce fruit and ultimately seed (Bhargava *et al.*, 2007b; Abdelbar, 2018). In open flowers, anthers are more spread than in closed flowers (Figure 8a), and flower opening may facilitate pollen dispersal, affecting the number of flowers that are fertilized. Consequently, if flowers remain closed under heat, fruit formation, seed number and yield may be impacted in quinoa. Previous studies in rice have shown that a closed flower is likely to be better protected from heat, but is less likely to be fertilized (Kobayasi *et al.*, 2010). Open and closed hermaphrodite flowers were counted from images of the main panicles acquired on day 6 of heat treatment. Images were captured between Zeitgeber time (ZT8) and ZT9, a time of day when approximately 50% of control treatment flowers were open. Shoot heating had a significant effect on the percentage of open hermaphrodite flowers (two‐way anova on the effect of shoot heating, *P* = 0.0001; Figure 8b). Root heating did not have a substantial impact on the percentage of open flowers (two‐way anova on the effect of root heating, *P* = 0.2552). HS plants had only 9.2% open hermaphrodite flowers and HRS plants had 15.2% open hermaphrodite flowers, whereas HR plants had 43.5% open hermaphrodite flowers and control plants had 57.3% open hermaphrodite flowers, on average (Wilcoxon rank sum test *P* values: control versus HS, *P* =0.00015; control versus HRS, *P* = 0.00035; control versus HR, *P* = 0.45145; Figure 8b). In summary, flowers remained closed in the heat treatments that ultimately had yield losses (HRS and HS), whereas flowers were open in the treatment (HR) with a yield similar to that of the control (Figure 8c). This result suggests that differences in flower opening during heat treatment could ultimately contribute to differences in yield.

## Conclusion

We explored the physiological changes that occur in quinoa accession QQ74 during and after heat treatment. The shoot heat treatments had the greatest number of physiological changes in response to heat. In particular, we find significant reductions in fruit production, delays in maturity, and increases in tertiary panicle yield and number in shoot treatments, suggesting that a heat treatment during anthesis triggers an avoidance strategy of prioritizing growth over development, with more resources allocated to tertiary panicles. The increase in tertiary panicle yield at the expense of primary and secondary panicle yield in heated‐shoot plants, compared with control plants, could be an effective survival strategy, but from an agricultural standpoint the yield from tertiary panicles is not likely to make a significant contribution to the total yield because quinoa is typically harvested when the main panicle has reached maturity. That said, as breeding programs commonly select against the development of tertiary and secondary panicles, so that more resources are dedicated to the main panicle, they may inadvertently impair heat‐stress tolerance mechanisms in quinoa. In the future, we are still very interested to test whether there are differences in seed quality among the treatments (e.g. amino acid profiles) and whether there are differences in quality from the different panicle types.

Gene expression data led us to examine floral phenotypes more closely, and we also observed a change in flower opening in the main panicle of HRS‐ and HS‐treated samples, with flowers remaining closed in the afternoon when control and HR samples had open flowers. This observation fits with the decreased fruit production in the main panicles of HRS and HS samples. Altogether, this work identifies key phenotypes to potentially mitigate heat‐induced yield decreases in quinoa. For example, if yield decreases in the main panicle are caused by floral closure induced by heat, then identifying natural variation or screening mutant populations for changes to these phenotypes might be targeted.

## Experimental Procedures

### Plant material and growth conditions

QQ74 (PI 614886) seeds were obtained from first‐generation (heterozygous) plants grown from the original US Department of Agriculture (USDA) seedstock. These seeds were planted in Pro‐Mix FPX (Premier Tech Horticulture, https://www.premiertech.com) soil and grown at 22°C, with a 12‐h photoperiod, 50% relative humidity and a light intensity of 400 µmol m^–2^ s^–1^. Plantlets at the developmental stage of between two and four leaves were transplanted to Berger BM7 soil (Berger, https://www.berger.ca) in 1.07 liter pots (T.O Plastics, Product 700051C, https://www.toplastics.com/), and grown under the same conditions. Before flowering, 60 of the plants most similar in height and developmental stage were selected and randomly divided into four treatment groups. Potted plants were placed in 245 cm × 68 cm × 16.5 cm wooden boxes filled with Turface Athletics MVP coarse sand (Profile Products LLC, https://www.profileproducts.com) to buffer soil temperature. The soil was covered with a blue mesh (Con‐Tact Brand, https://contactbrand.com) to facilitate image processing. When more than 50% of the plants had open flowers (35–40 days after planting), all plants were moved to a secondary growth chamber with the same conditions for 2 days, while the temperature was adjusted and stabilized for heat treatments.

### Heat treatments

Four treatment groups were used in this experiment: (i) control, with plants growing at 22°C; (ii) heated roots, with roots growing at 30°C and shoots growing at 22°C; (iii) heated shoots, with roots growing at 22°C and shoots growing at 35°C; and (4) heated roots and shoots, with roots growing at 30°C and shoots growing at 35°C (Figure S1). For setting up the four treatments, the wooden boxes were split into two growth chambers. The first growth chamber was set to control conditions of 22°C, with a 12‐h photoperiod, 50% relative humidity and 400 µmol m^–2^ s^–1^ light intensity; the second growth chamber was set to 35°C, with a 12‐h photoperiod, 50% relative humidity and 400 µmol m^–2^ s^–1^ light intensity. In the first growth chamber, a heating coil at 30°C was passed through the sand of one wooden box at two heights, approximately equally spaced around the pots, to heat the soil to 30°C (HR). In the second growth chamber held at the higher temperature, a cool water line running 15.5°C water was passed through the sand of one wooden box at two heights, approximately equally spaced around the pots to cool the soil to 22°C (HS; Figure S1). At ZT0, plants were divided into four treatments, where each treatment was contained in a wooden box. The heat treatment lasted 11 days. After 11 days of treatment, plants were moved for a day to a third growth chamber at control conditions while the temperature was adjusted back to control conditions in the treatment chambers. Once control conditions (22°C) were reached in the treatment chambers, plants were replaced in their respective wooden boxes at control conditions, for 7–10 more days, and subsequently moved to a glasshouse with control conditions until harvest for yield measurements.

### Sampling for pollen viability, fresh weight, and dry weight

Samples from two to four plants per treatment were collected between ZT2 and ZT4, at days 1 and 11 of heat treatment. Flowers with visible anthers were cut and placed in Alexander stain (Alexander, 1969) for pollen viability assays. Sampled plants were cut to separate the shoots from the roots. Shoots were immediately weighed to obtain fresh weight, and subsequently both roots and shoots were dried at 40°C and 30% relative humidity for 3 days and weighed to obtain the dry weight.

### Seed harvesting

When the plants stopped water uptake from the soil, the watering stopped and the plants were allowed to dry until they were ready to harvest. Shoots and panicles were cut and stored in paper bags at room temperature (approximately 23°C). Seed was harvested from the shoots using an air‐blast seed cleaner (ABSC; Almaco, https://www.almaco.com). Seed was then manually cleaned with a mesh and stored in paper envelopes at room temperature.

### Main panicle, seed and whole‐plant imaging

A Raspberry Pi computer (https://www.raspberrypi.org) controlling a single‐lens reflex (SLR) camera (Nikon COOLPIX L830, https://www.nikon.com) on a camera stand was used to collect main panicle and seed images at harvest (https://doi.org/10.5281/zenodo.3352280). The camera set‐up to collect main panicle and seed images was described in detail by Tovar *et al. *(2018: appendix 3). The main panicle images were analyzed using plantcv (Gehan *et al.*, 2017) to measure the main panicle area, width, height, hull area, solidity, perimeter, longest axis, and hull vertices. The python script analyze_image.py (https://github.com/danforthcenter/quinoa-heat-tovar/blob/master/analyze_image.py) was run over all images using the PlantCV script pantcv‐pipeline.py. The r script used to analyze the main panicle output measurements is available from Github (https://github.com/danforthcenter/quinoa-heat-tovar/blob/master/main_panicle_image_data_analysis.R). To estimate seed size, a subset of quinoa seeds collected from control, HR, HS, and HRS plants were imaged and then that subset of seeds was weighed. The number of seeds produced per plant was estimated by dividing the total seed yield of every plant by the estimated individual seed weight of the same plant. A 1.27 cm tough spot was used as a size marker for seed images. Images were analyzed using plantcv (Gehan *et al.*, 2017) to measure seed area. An example jupyter notebook for seed image processing (https://github.com/danforthcenter/quinoa-heat-tovar/blob/master/seed-image-analysis/plantcv-seed-phenotyping-phenomatics.ipynb) and the r script used to process seed area results (https://github.com/danforthcenter/quinoa-heat-tovar/blob/master/seed-image-analysis/heat-seed-quinoa-analysis.R) are available from Github. Adjustments to the region of interest were made for different seed images, to make sure that all seed and size markers were included in the analysis.

Whole‐plant images and images of main panicles during heat treatment were acquired using the same SLR camera used for seed imaging, with white poster boards as a background (https://doi.org/10.5281/zenodo.3352280). To manually count open and closed hermaphrodite flowers, images of main panicles were acquired on day 6 of heat treatment. Whole‐plant images were acquired 45 days after the end of heat treatment (approximately 95 days after planting). An example jupyter notebook used to analyze whole‐plant images (https://github.com/danforthcenter/quinoa-heat-tovar/blob/master/quinoa_heat_whole_plants.ipynb) and the probability density functions file (https://github.com/danforthcenter/quinoa-heat-tovar/blob/master/whole_plant_pdfs.txt) used to classify green and yellow plant parts are also available on Github. Adjustments to the placement of black boxes, thresholds, and the region of interest were made in the jupyter notebook when analyzing whole‐plant images, to make sure that the entire plant was included in the analysis. The side of the pot was measured in pixels using imagej (Schneider *et al.*, 2012) for each whole‐plant image, and was used to normalize linear measurements (perimeter, height, width and longest axis), whereas the square of the pot side was used to normalize area measurements (area, and hull area). The r script used to analyze whole‐plant image measurements is available from Github (https://github.com/danforthcenter/quinoa-heat-tovar/blob/master/whole_plant_images.R).

### RNA‐Seq

Samples were collected from three plants per treatment at four time points: 1, 2 and 11 days of heat treatment, and 1 day after heat treatment ended. Each plant was considered a biological replicate. Leaf tissue, root tissue and flower tissue was collected at ZT2 and immediately frozen in liquid nitrogen. Although four time points and multiple tissue types were collected, only leaf samples from day 1 and day 11 were processed further for RNA‐seq. Total RNA was extracted from quinoa leaves with the RNeasy Plant Mini Kit, including the RNase‐free DNase Set, as recommended by the manufacturer (Qiagen, https://www.qiagen.com). RNA was quantified with Qubit RNA BR Assay Kit (Invitrogen, now ThermoFisher Scientific, https://www.thermofisher.com), and quality was assessed with the Agilent 6000 Nano Kit (Agilent, https://www.agilent.com). Strand‐specific library construction and paired‐end RNA sequencing were performed by Novogene using Illumina HiSeq 4000 (Novogene, https://en.novogene.com). The resulting paired‐end reads were analyzed for quality using fastqc 0.11.7 (Andrews *et al.*, 2012), where the 'per base sequence content' showed the first 10–20 bases on the 5′ ends contained low‐quality reads, suggesting the need to trim those bases. Based on fastqc quality analysis, reads were trimmed using trimmomatic 0.38 (Bolger *et al.*, 2014) with phred 33 quality scores (option '‐phred33'), removing adapters (option 'ILLUMINACLIP:TruSeq3‐PE.fa:2:30:10'), cropping 15 bases from the 5′ end (option 'HEADCROP:15'), and using the default options 'LEADING:3 TRAILING:3 SLIDINGWINDOW:4:15 MINLEN:36'. After read trimming, the 'per base sequence content' in fastqc showed good quality, indicating that the reads were ready for gene expression profiling. A quinoa transcriptome index was created with kallisto 0.44.0 (Bray *et al.*, 2016), using the 'kallisto index' command with required argument '‐' and no optional arguments, from the published quinoa transcriptome (file Cquinoa_392_v1.0.transcript.fa.gz) (Jarvis *et al.*, 2017) available at Phytozome (https://phytozome.jgi.doe.gov; Goodstein *et al.*, 2012). Transcript abundance was quantified with kallisto 0.44.0 (Bray *et al.*, 2016) by pseudo aligning the trimmed paired‐end reads to the quinoa transcriptome index created, using the 'kallisto quant' command with required arguments '‐' and '‐o', and optional bootstrapping set to 100 (argument '‐b'). Differential analysis of transcript levels between treatments and control was performed with sleuth 0.30.0 (Pimentel *et al.*, 2017) in rstudio 1.1.463 and r 3.5.2. A *q* < 0.05 was used to call differentially expressed genes. The raw RNA‐seq reads and the relative gene expression levels resulting from differential expression analysis with sleuth are available from the National Center for Biotechnology Information (NCBI) Gene Expression Omnibus (GEO) submission GSE128155. Read statistics are described in Table S2. The r script rna_seq_quinoa_heat.R used to analyze differential gene expression is available at https://github.com/danforthcenter/quinoa-heat-tovar/blob/master/rna_seq_quinoa_heat.R.

### Gene ontology analysis

Gene ontology (GO) was performed on differentially expressed genes found in RNA‐seq analysis. panther 14.0 (http://pantherdb.org) was used for GO analysis (Mi *et al.*, 2013). Differentially expressed gene lists were associated with their panther IDs from the quinoa genome (v1.0) annotation (Jarvis *et al.*, 2017), available at Phytozome (Goodstein *et al.*, 2012). Text files with gene lists were uploaded into panther using the 'PANTHER Generic Mapping' option, following panther recommended file formatting, with quinoa gene names on the first column, and panther IDs on the second column, and using 'NOHIT' whenever a gene did not have an associated panther ID. The b values obtained from differential expression analysis with sleuth 0.30.0 were used as the third column (numerical value of the experiment) for statistical enrichment tests. Statistical over‐representation and enrichment tests were performed using Fisher's exact test and false discovery rate (FDR) *P* correction on all three GO categories: molecular function, biological process and cellular component. For statistical over‐representation tests, a text file containing all genes in the quinoa genome as the first column and their respective panther IDs in the second column was uploaded and used as a reference list. The r script GO_analysis_quinoa_heat.R used to produce the files used for GO analysis is available at https://github.com/danforthcenter/quinoa-heat-tovar/blob/master/GO_analysis_quinoa_heat.R.

### Transcription factor homolog identification

Differentially expressed genes obtained from RNA‐seq analysis were screened to identify homologs of *Arabidopsis thaliana* transcription factors. *Arabidopsis thaliana* orthologs corresponding to each quinoa gene were obtained from the quinoa genome annotation (Jarvis *et al.*, 2017) available in Phytozome (Goodstein *et al.*, 2012). To identify quinoa transcription factor homologs, the list of quinoa genes and their respective *A. thaliana* gene homolog names were cross‐referenced with the list of Arabidopsis transcription factors available at http://planttfdb.cbi.pku.edu.cn (Jin *et al.*, 2017). The r script used to identify transcription factor homologs is included in the rna_seq_quinoa_heat.R r script, available at https://github.com/danforthcenter/quinoa-heat-tovar/blob/master/rna_seq_quinoa_heat.R.

### Statistical analysis

Data were analyzed in rstudio 1.1.463, with r 3.5.2. A *P* < 0.05 was considered statistically significant. Each plant was considered a biological replicate. As data were not normally distributed and did not have homogeneous variances across treatments, non‐parametric analyses were performed. Curves were analyzed using a Kolmogorov–Smirnov test (Kolmogorov, 1933; Smirnov, 1948) with the ks.test function (Figures 4a,b, S2a and S3). Individual time points were analyzed with a robust two‐way anova using the med2way function from the wrs2 package (https://r-forge.r-project.org/projects/psychor/), with factors 'root heating' and 'shoot heating'. When a statistical effect was found with the anova, a Kruskal–Wallis test (Kruskal and Wallis, 1952) was performed with the kruskal.test function to confirm that differences existed among treatments. If differences were confirmed by Kruskal–Wallis test, a pairwise Wilcoxon rank sum test (Mann and Whitney, 1947) using the pairwise.wilcox.test function with a Benjamini and Yekutieli *P* correction (Benjamini and Yekutieli, 2001) (argument p.adjust.method = 'BY') was used to find which treatment was different from which, and the respective *P *values (Figures 1, 2, 5, 6 and S2b). Analysis of individual secondary panicle yield was performed through anovas with factors: 'shoot heating', 'root heating' and 'panicle position' (Figure 3a), or 'shoot heating', 'root heating' and 'length of heat treatment' (Figure 3b). For RNA‐seq data analysis (Figure 7), please see the RNA‐seq section of the Experimental procedures.

## Conflict of Interest

The authors declare that they have no conflicts of interest.

## Author Contributions

JT, SC and MG conceived and designed the research; SC designed and prepared sandboxes for differential root or shoot heating; JT designed the experiments; JT, CQ, EC, PP and AS performed the experiments; JT, HS, and MG analyzed the data; MG developed the quinoa heat data explorer app in r shiny; JT and MG wrote the article. JT, SC, HS, NF and MG edited the article.

### Open Research Badge

This article has earned an Open Data Badge for making publicly available the digitally‐shareable data necessary to reproduce the reported results. The data is available at https://doi.org/10.5281/zenodo.3352281; https://github.com/danforthcenter/quinoa-heat-tovar.

## Supporting information


**Figure S1. **Image of sandbox system to apply heat and cooling treatments.

**Figure S2.** Photosystem‐II efficiency was not changed by heat treatment.

**Figure S3.** Pollen viability measured during days 1 and 11 of heat treatment.

**Figure S4.** Shoot fresh weight and water content were not modified after heat treatment.

**Figure S5.** Root dry weight was not substantially affected by heat treatment.

**Table S1.** List of 10 transcription factors that were differentially expressed in both HRS and HS treatments during both days 1 and 11 of heat treatment.

**Table S2.** Read statistics for all RNA‐seq samples.

## Data Availability

All data from this study is publicly available at https://doi.org/10.5281/zenodo.3352280. Specific locations for each data set are mentioned in the Experimental procedures section.
