# Supplementary figures and images for: Heating quinoa shoots results in yield loss by inhibiting fruit production and delaying maturity
Source: Plant J. 2020 Feb 24;102(5):1058–73. doi: 10.1111/tpj.14699 (PMC7318176; doi:10.1111/tpj.14699)

**a.**

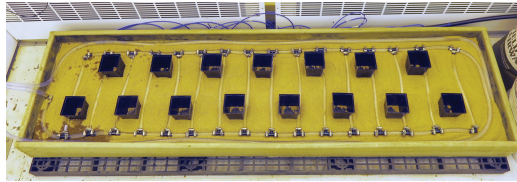

**b.**

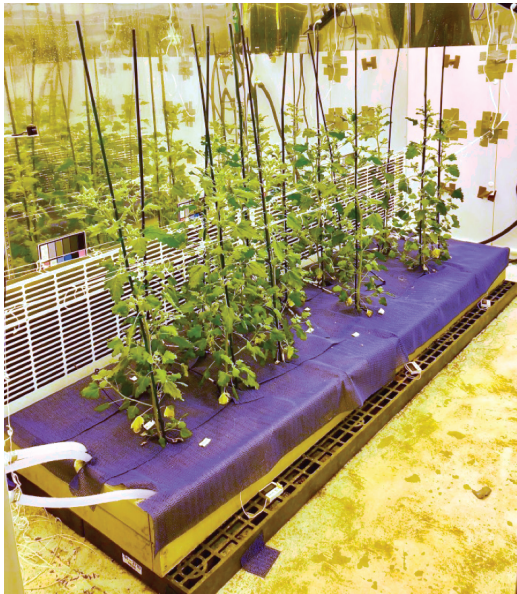

Supplement: Supplementary file 1 — Figure S1. Image of sandbox system to apply heat and cooling treatments. [file TPJ-102-1058-s001.pdf]

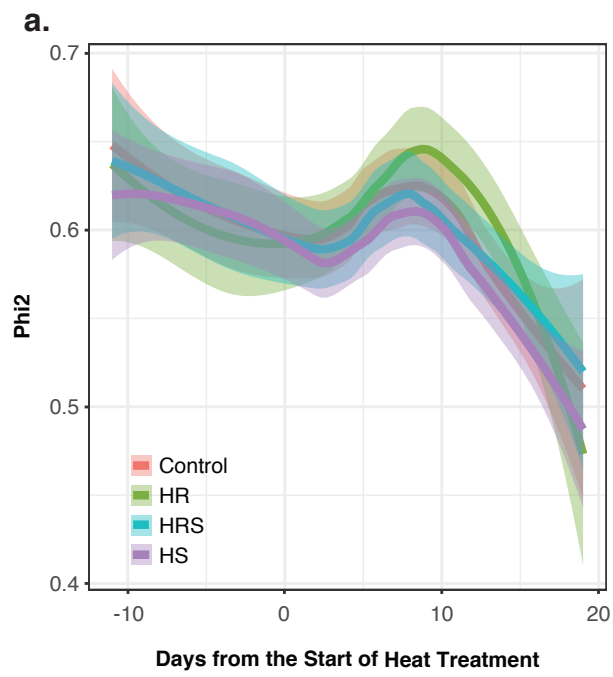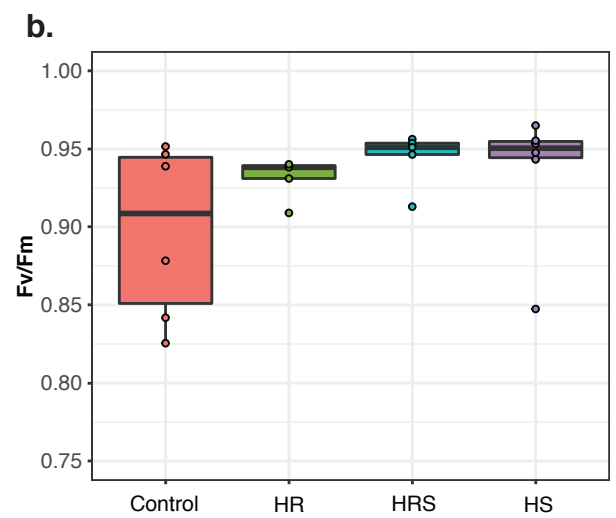

Supplement: Supplementary file 2 — Figure S2. Photosystem‐II efficiency was not changed by heat treatment. [file TPJ-102-1058-s002.pdf]

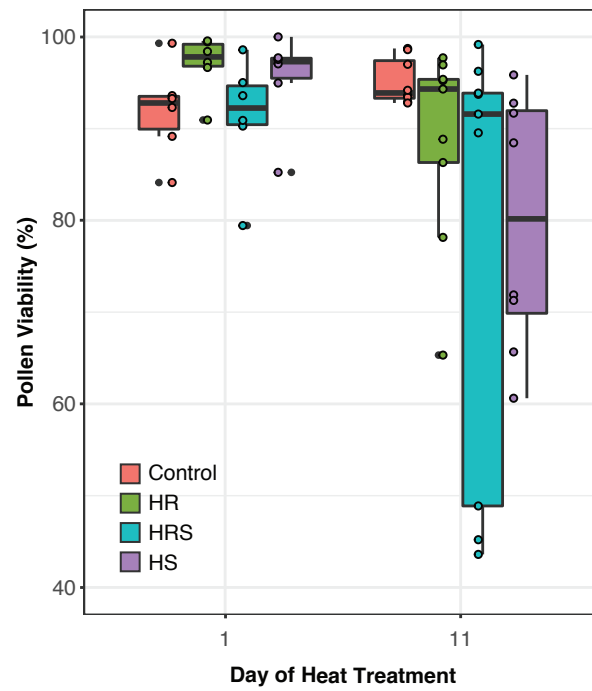

Supplement: Supplementary file 3 — Figure S3. Pollen viability measured during days 1 and 11 of heat treatment. [file TPJ-102-1058-s003.pdf]

**a.**

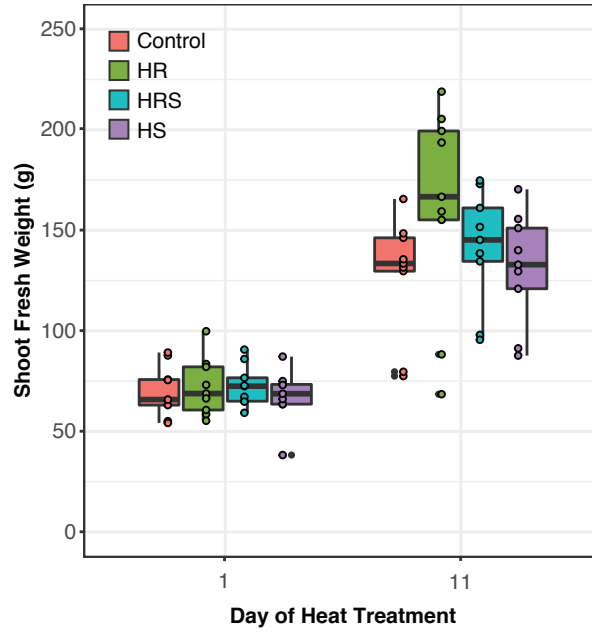

**b.**

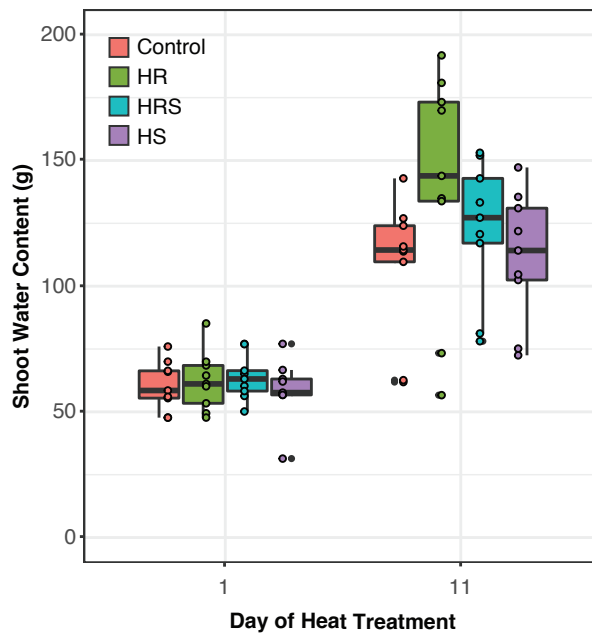

Supplement: Supplementary file 4 — Figure S4. Shoot fresh weight and water content were not modified after heat treatment. [file TPJ-102-1058-s004.pdf]

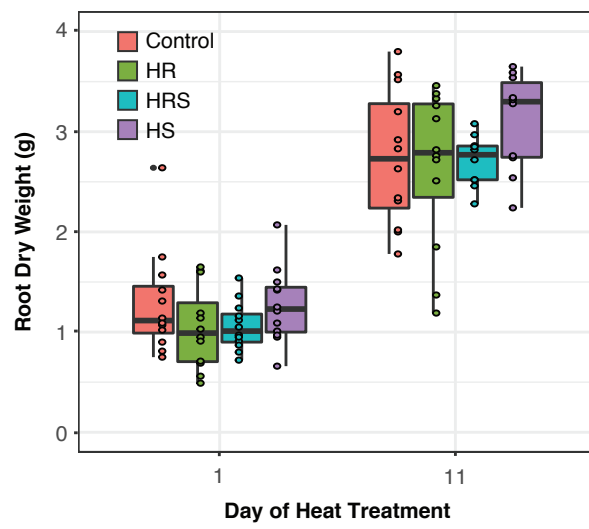

Supplement: Supplementary file 5 — Figure S5. Root dry weight was not substantially affected by heat treatment. [file TPJ-102-1058-s005.pdf]
