# Supplementary material for: Heating quinoa shoots results in yield loss by inhibiting fruit production and delaying maturity
Source: Plant J. 2020 Feb 24;102(5):1058–73. doi: 10.1111/tpj.14699 (PMC7318176; doi:10.1111/tpj.14699)
Supplement: Supplementary file 6 — Table S1. List of 10 transcription factors that were differentially expressed in both HRS and HS treatments during both days 1 and 11 of heat treatment. [file TPJ-102-1058-s006.pdf]

Quinoa Gene	Arabidopsis Homolog	Arabidopsis Gene Name	Gene Symbol	HRS b for Day 1	HS b for Day 1	HRS b for Day 11	HS b for Day 11
AUR62001266-RA	AT3G48440.1	Zinc finger CCCH domain-containing protein 43	At3g48440	-0.4767773743	-0.4639581859	-0.4323383341	-0.4405534753
AUR62004822-RA	AT3G04450.1	Myb family transcription factor PHL13	PHL13	-0.7364080574	-0.7378122409	-0.7345801281	-0.6691453536
AUR62008425-RA	AT3G47500.1	Cyclic dof factor 3	CDF3	-0.270388337	-0.2418169937	-0.4642102431	-0.4395060492
AUR62012948-RA	AT2G17040.1	NAC domain containing protein 36	NAC036	-2.454984848	-3.25263294	-1.909195261	-1.511841101
AUR62016149-RA	AT1G10240.1	Protein FAR1-RELATED SEQUENCE 11	FRS11	1.177639699	1.101948002	1.41982516	1.415349134
AUR62019043-RA	AT4G24540.1	MADS-box protein AGL24	AGL24	-0.2620954991	-0.3422856085	-0.6746297196	-0.9076483211
AUR62021459-RA	AT4G29100.1	Transcription factor bHLH68	BHLH68	-0.8220734518	-0.545311355	-0.7614201284	-0.8632236947
AUR62033383-RA	AT4G11880.1	Agamous-like MADS-box protein AGL14	AGL14	-0.5775236714	-0.2885525882	-0.4649410572	-0.5857004328
AUR62034763-RA	AT5G62000.2	Auxin response factor 2	ARF2	-0.5785349602	-0.3591114046	-0.4468921575	-0.5011076893
AUR62039063-RA	AT3G19500.1	Transcription factor bHLH113	BHLH113	1.458845177	1.69610382	1.196139371	1.40084197
