# Supplementary material for: Heating quinoa shoots results in yield loss by inhibiting fruit production and delaying maturity
Source: Plant J. 2020 Feb 24;102(5):1058–73. doi: 10.1111/tpj.14699 (PMC7318176; doi:10.1111/tpj.14699)
Supplement: Supplementary file 7 — Table S2. Read statistics for all RNA‐seq samples. [file TPJ-102-1058-s007.pdf]

Day of Heat Treatment Sampled	Treatment	Plant Number	Sample Name	Number of Reads	Mapped Reads	Unique Reads	% Pseudoaligned Reads	% Unique Reads	Number of Targets
1	control	20	C20m1	16489067	13314790	12160190	80.7	73.7	44776
1	control	22	C22m1	20448026	16435986	15135340	80.4	74	44776
1	control	27	C27m1	17738071	14165473	13361796	79.9	75.3	44776
1	HR	7	HR7m1	18781898	15178535	13963839	80.8	74.3	44776
1	HR	9	HR9m1	13048505	10512223	9624442	80.6	73.8	44776
1	HR	14	HR14m1	22954259	18801943	17383223	81.9	75.7	44776
1	HRS	51	HRS51m1	18966994	15346565	14266518	80.9	75.2	44776
1	HRS	55	HRS55m1	20090463	16212643	15097973	80.7	75.1	44776
1	HRS	58	HRS58m1	19422866	15296035	14354188	78.8	73.9	44776
1	HS	35	HS35m1	23342760	18899300	17646604	81	75.6	44776
1	HS	39	HS39m1	17998525	14540310	13742856	80.8	76.4	44776
1	HS	43	HS43m1	26157723	21218211	20100293	81.1	76.8	44776
11	control	16	C16m11	20438725	17135528	16291574	83.8	79.7	44776
11	control	28	C28m11	22828239	18918366	17443407	82.9	76.4	44776
11	control	30	C30m11	22961722	19091043	18040050	83.1	78.6	44776
11	HR	1	HR1m11	18170857	15067185	14361808	82.9	79	44776
11	HR	5	HR5m11	20346428	16953406	16071979	83.3	79	44776
11	HR	11	HR11m11	24596593	20466759	19429077	83.2	79	44776
11	HRS	47	HRS47m11	18238913	14961804	14077716	82	77.2	44776
11	HRS	54	HRS54m11	15513950	12877264	11724210	83	75.6	44776
11	HRS	59	HRS59m11	21346979	17509601	16277675	82	76.3	44776
11	HS	31	HS31m11	26814025	21751160	19525837	81.1	72.8	44776
11	HS	34	HS34m11	19001964	15083846	13863121	79.4	73	44776
11	HS	41	HS41m11	15607647	12498187	11147137	80.1	71.4	44776
