# Supplementary material for: Heating quinoa shoots results in yield loss by inhibiting fruit production and delaying maturity
Source: Plant J. 2020 Feb 24;102(5):1058–73. doi: 10.1111/tpj.14699 (PMC7318176; doi:10.1111/tpj.14699)
Supplement: Supplementary file 8 [file TPJ-102-1058-s008.docx]

**Supporting Methods**

*Photosynthetic measurements*

Photosynthetic rates were measured with a MultispeQ (version 1.0, PhotosynQ, East Lansing, Michigan), using the Leaf Photosynthesis MultispeQ V1.0 (<http://photosynq.org/protocols/leaf-photosynthesis-multispeq-v1-0>, [(Kuhlgert *et al.*, 2016)](https://paperpile.com/c/8tRQJt/g3sS)) and The One v3.0 (<http://photosynq.org/protocols/the-one-v3-0-phi2-npqt-using-multi-phase-flash-with-qi-qe-phi2no-rg>) protocols. MultispeQ measurements were taken on 3 plants per treatment, every day during the 11 days of heat treatment, between ZT8 and ZT10. Leaves of the same developmental age were measured every day. Fv/Fm was measured on day 11 of heat exposure, using a Li-cor LI-6400XT (Li-cor, Nebraska, USA) following manufacturer’s recommendations.

*Pollen viability*

At least two anthers from different flowers of the same plant were manually opened to release pollen into 10 µl of Alexander stain [(Alexander, 1969)](https://paperpile.com/c/8tRQJt/olql). Pollen was stained for 15 minutes and then centrifuged at 20,000 x g for 1 minute to spin pollen down. To concentrate pollen in the stain, the top 5 µl of stain were discarded. Pollen was resuspended in the remaining 5 µl of stain and analyzed for viability under a brightfield microscope (Omax, Gyeonggi-do, Korea). At least 100 pollen grains were counted per plant to estimate percentage viability, considering green-stained pollen as non-viable, and red or purple-stained pollen as viable [(Alexander, 1969)](https://paperpile.com/c/8tRQJt/olql).

**Supporting Information**

*Photosystem II efficiency of heat treated plants was similar to control treatment before, during, and after heat treatment.*

To assess the effect of heat on photosystem II efficiency in quinoa, Phi2, a measurement of the quantum yield of photosystem II, was measured using a MultispeQ (v1.0) [(Kuhlgert *et al.*, 2016)](https://paperpile.com/c/8tRQJt/g3sS). Measurements were taken from 11 days before heat treatment started, during the 11 days of heat treatment, and 8 days after heat treatment ended. Analysis of Phi2 data using the Kolmogorov-Smirnov test showed that the heated shoots treatment was different from control (p-values 0.003688, Figure S2a). To verify the differences in photosystem II efficiency measured with Phi2, Fv/Fm was used as an independent measurement of the quantum yield of photosystem II. Fv/Fm was measured on day 11 of heat treatment, when we might expect the impact of the progressive heat treatment to be the greatest [(Becker *et al.*, 2017; Yang *et al.*, 2016)](https://paperpile.com/c/8tRQJt/oj9B+36KH). However, there was no substantial effect of heat on Fv/Fm in any treatment (4 to 5% average change from control, Kruskal-Wallis test p-value 0.09369, Figure S2b). Similarly, Hinojosa et al. 2018 [(Hinojosa, Matanguihan, *et al.*, 2018)](https://paperpile.com/c/8tRQJt/8IGR) also did not find substantial changes from heat on Fv/Fm in quinoa accession QQ74. A different study in quinoa cultivar Titicaca found that plants grown at 25/20°C (day/night) had higher Fv/Fm than plants grown at 18/8°C (day/night) [(Yang *et al.*, 2016)](https://paperpile.com/c/8tRQJt/36KH), but the yield from these plants was not reported. Overall, there was no conclusive evidence to support changes in photosystem II efficiency due to heat in quinoa. This likely indicates that photosystem II is not a substantial factor in yield losses of quinoa from heat.

*Pollen viability after heat treatment was similar to control treatment*

The observed low fruit production after shoot heating could be a result of lower pollen viability under heat treatment, therefore pollen viability was measured. Pollen viability was assessed at the start and end of heat treatments by measuring the rate of pollen abortion through Alexander staining [(Alexander, 1969)](https://paperpile.com/c/8tRQJt/olql). Pollen viability as measured by pollen abortion in shoot or root heated samples did not show evidence of being substantially affected as compared to control (2 to 11% average change from control, Kolmogorov-Smirnov test p-values > 0.05, Figure S3). Since heat did not seem to substantially affect the rate of aborted pollen, this would suggest that the observed low fruit production after shoot heating is unlikely due to changes in pollen viability. Interestingly, Hinojosa et al. 2018 [(Hinojosa, Matanguihan, *et al.*, 2018)](https://paperpile.com/c/8tRQJt/8IGR) found heat resulted in 63% lower pollen viability than control, in the same quinoa cultivar used in this study (QQ74). However a different pollen staining method (tetrazolium), was used [(Hinojosa, Matanguihan, *et al.*, 2018)](https://paperpile.com/c/8tRQJt/8IGR). Interestingly, the significantly lower pollen viability in Hinojosa et al. 2018 did not affect yield [(Hinojosa, Matanguihan, *et al.*, 2018)](https://paperpile.com/c/8tRQJt/8IGR). Since no differences in pollen viability were found from heat treatment, and the study of Hinojosa et al. 2018 [(Hinojosa, Matanguihan, *et al.*, 2018)](https://paperpile.com/c/8tRQJt/8IGR) found that a dramatically lower pollen viability did not affect yield, this would suggest that pollen viability is not the main limitation for quinoa fruit and seed production. However, the different pollen viability results of this study and of Hinojosa et al. 2018 [(Hinojosa, Matanguihan, *et al.*, 2018)](https://paperpile.com/c/8tRQJt/8IGR) highlight the importance of the method used to measure pollen viability. A more definitive method for measuring pollen viability is in vitro pollen germination [(Sato *et al.*, 2000; Hinojosa, Matanguihan, *et al.*, 2018)](https://paperpile.com/c/8tRQJt/Eozl+8IGR), but to our knowledge, there is no published in vitro pollen germination protocol for quinoa [(Hinojosa, Matanguihan, *et al.*, 2018)](https://paperpile.com/c/8tRQJt/8IGR). Pollen viability studies through in vitro germination would provide more conclusive evidence to whether pollen viability is affected by heat treatment in quinoa, and its potential impact in fruit and seed production.

*Shoot fresh weight did not show substantial changes during heat treatment*

To assess the effect of heat on shoot biomass, shoot fresh weight was measured on the first and last days of heat treatment. At the start of heat treatment shoot fresh weight was very similar among all plants (Kruskal-Wallis test p-value 0.9465). On the last day of heat treatment, shoot fresh weight had not substantially changed in comparison to the control treatment (Kruskal-Wallis test p-value 0.1226, Figure S4a). To assess differences in water content, the amount of water in each plant was also calculated by subtracting the shoot dry weight from the shoot fresh weight of each plant. As with fresh weight, no substantial differences in water content were found neither on the first (Kruskal-Wallis test p-value 0.8428) nor on the last day of heat (Kruskal-Wallis test p-value 0.1086, Figure S4b). Since it was expected that heat would increase water demands for treated plants, plants were well watered according to demand to prevent drought stress. Thus, quinoa was able to retain control water levels even during heat treatment.

*Root dry weight did not show substantial changes during heat treatment*

Root dry weight was measured on the first and last days of heat treatment to assess the effects of heat on root biomass. There were no substantial differences in root dry weight, both at the start (Kruskal-Wallis test p-value 0.3462) and end of heat treatment (Kruskal-Wallis test p-value 0.3351, Figure S5). The study of Hinojosa et al., 2018 [(Hinojosa, Matanguihan, *et al.*, 2018)](https://paperpile.com/c/8tRQJt/8IGR) also found no substantial differences in root dry weight 8 days after heat treatment, and therefore also suggests that heat does not affect root biomass in quinoa. Although there was no indication of changes in root dry weight from heat, in both studies, plants were grown in pots, which could have limited root growth. Measurements of root dry weight from heat-treated plants grown in the field would provide a more conclusive assessment of root dry weight changes after heat.

*Quinoa Heat Data Explorer: a tool for navigating and analyzing quinoa differential gene expression under heat*

Quinoa Heat Data Explorer is a tool built to facilitate sharing and analysis of the gene expression data obtained in this study with the quinoa research community. Quinoa Heat Data Explorer can be accessed at <http://shiny.datasci.danforthcenter.org/quinoa-heat/>, or downloaded from <https://github.com/danforthcenter/quinoa-heat-tovar> and run locally using R or RStudio. With the Quinoa Heat Data Explorer tool, users can browse and search differentially expressed quinoa genes using significance level cutoffs (q-values). Alternatively, users can search for GO terms, or ortholog *Arabidopsis thaliana* gene IDs. Gene expression graphs can be generated and graphs and data can be downloaded directly from the tool. This tool can be expanded to include new quinoa expression data as it becomes available.

Quinoa Heat Data Explorer was developed using Shiny (<http://shiny.rstudio.com/>). The code for Quinoa Heat Data Explorer is available at <https://github.com/danforthcenter/quinoa-heat-tovar>. The complete list of genes in the quinoa genome was associated with their respective *A. thaliana* orthologs available from the Phytozome [(Goodstein *et al.*, 2012)](https://paperpile.com/c/8tRQJt/SJnv) quinoa genome annotation [(Jarvis *et al.*, 2017)](https://paperpile.com/c/8tRQJt/QwIr). The list of GO terms corresponding to each quinoa gene was obtained by submitting the list of all *A. thaliana* orthologs to Panther (<http://pantherdb.org/>) [(Mi *et al.*, 2013)](https://paperpile.com/c/8tRQJt/pCGD) and retrieving the corresponding GO terms. For each gene, the corresponding corrected differential expression significance value (q value) and the expression value (b value, labelled “de” in Quinoa Heat Data Explorer) as obtained from analysis with Sleuth was added for each treatment and day of heat treatment sampled.

**Supporting Table Legends**

**Table S1.** List of ten genes that were differentially expressed in both HRS and HS treatments during both days 1 and 11 of heat treatment, and were also identified as homologous to *A. thaliana* transcription factors. b values represent the effect size of the treatment on transcript abundance [(Pimentel *et al.*, 2017)](https://paperpile.com/c/2netUO/fLpZ).

**Table S2.** Read statistics for all RNA-seq samples.

**Supporting Figure Legends**

**Figure S1.** Sandbox system to apply heat and cooling treatments. a) Sandbox system with cooling hose running around pots. b) Sandbox system with quinoa plants during heat treatment.

**Figure S2.** Photosystem II efficiency was not changed by heat treatment. a) Phi2 measured from 10 days before heat treatment started, during heat treatment, and until 8 days after heat treatment ended (n = 3 to 8 plants per timepoint and per treatment). Curves resulting from a LOESS polynomial regression are shown. b) Fv/Fm measured after 11 days in heat treatment per plant for each treatment (n =5 for control and HS; n=6 for HR and HRS).

**Figure S3.** Pollen viability measured during 1 and 11 days of heat treatment (n=6 plants per treatment for day 1, and n=8 plants per treatment for day 11).

**Figure S4.** Shoot fresh weight and water content were not modified after heat treatment. a) Shoot fresh weight measured from each plant and for each treatment (n=9). b) Shoot water content measured from each plant and for each treatment (n=9).

**Figure S5.** Root dry weight was not substantially affected by heat treatment. Root dry weight measured for each plant and for each treatment at 1 and 11 days of heat treatment (n=12).
