# Supplementary material for: Heating quinoa shoots results in yield loss by inhibiting fruit production and delaying maturity
Source: Plant J. 2020 Feb 24;102(5):1058–73. doi: 10.1111/tpj.14699 (PMC7318176; doi:10.1111/tpj.14699)
Supplement: Supplementary file 9 [file TPJ-102-1058-s009.pdf]

Supporting Table Legends

Table S1. List of ten genes that were differentially expressed in both HRS and HS treatments during both days 1 and 11 of heat treatment, and were also identified as homologous to *A. thaliana* transcription factors. b values represent the effect size of the treatment on transcript abundance (Pimentel *et al.*, 2017).

Table S2. Read statistics for all RNA-seq samples.

Supporting Figure Legends

Figure S1. Sandbox system to apply heat and cooling treatments. a) Sandbox system with cooling hose running around pots. b) Sandbox system with quinoa plants during heat treatment.

Figure S2. Photosystem II efficiency was not changed by heat treatment. a) Phi2 measured from 10 days before heat treatment started, during heat treatment, and until 8 days after heat treatment ended (n = 3 to 8 plants per timepoint and per treatment). Curves resulting from a LOESS polynomial regression are shown. b) Fv/Fm measured after 11 days in heat treatment per plant for each treatment (n =5 for control and HS; n=6 for HR and HRS).

Figure S3. Pollen viability measured during 1 and 11 days of heat treatment (n=6 plants per treatment for day 1, and n=8 plants per treatment for day 11).

Figure S4. Shoot fresh weight and water content were not modified after heat treatment. a) Shoot fresh weight measured from each plant and for each treatment (n=9). b) Shoot water content measured from each plant and for each treatment (n=9).

Figure S5. Root dry weight was not substantially affected by heat treatment. Root dry weight measured for each plant and for each treatment at 1 and 11 days of heat treatment (n=12).
